# Biochemical and Biophysical Characterization of Recombinant Human 3-Phosphoglycerate Dehydrogenase

**DOI:** 10.3390/ijms22084231

**Published:** 2021-04-19

**Authors:** Giulia Murtas, Giorgia Letizia Marcone, Alessio Peracchi, Erika Zangelmi, Loredano Pollegioni

**Affiliations:** 1 Department of Biotechnology and Life Sciences, University of Insubria, via J.H. Dunant 3, 21100 Varese, Italy; letiziamarcone@alice.it (G.L.M.); loredano.pollegioni@uninsubria.it (L.P.); 2 Department of Chemistry, Life Sciences and Environmental Sustainability, University of Parma, 43124 Parma, Italy; alessio.peracchi@unipr.it (A.P.); erika.zangelmi@unipr.it (E.Z.)

**Keywords:** L-serine, D-serine, NAD, biochemical properties, metabolism, NMDA receptor, neurotransmission, cancer progression, phosphorylated pathway, serine deficiency

## Abstract

The human enzyme D-3-phosphoglycerate dehydrogenase (hPHGDH) catalyzes the reversible dehydrogenation of 3-phosphoglycerate (3PG) into 3-phosphohydroxypyruvate (PHP) using the NAD^+^/NADH redox cofactor, the first step in the phosphorylated pathway producing L-serine. We focused on the full-length enzyme that was produced in fairly large amounts in *E. coli* cells; the effect of pH, temperature and ligands on hPHGDH activity was studied. The forward reaction was investigated on 3PG and alternative carboxylic acids by employing two coupled assays, both removing the product PHP; 3PG was by far the best substrate in the forward direction. Both PHP and α-ketoglutarate were efficiently reduced by hPHGDH and NADH in the reverse direction, indicating substrate competition under physiological conditions. Notably, neither PHP nor L-serine inhibited hPHGDH, nor did glycine and D-serine, the coagonists of NMDA receptors related to L-serine metabolism. The investigation of NADH and phosphate binding highlights the presence in solution of different conformations and/or oligomeric states of the enzyme. Elucidating the biochemical properties of hPHGDH will enable the identification of novel approaches to modulate L-serine levels and thus to reduce cancer progression and treat neurological disorders.

## 1. Introduction

**Figure 1:**
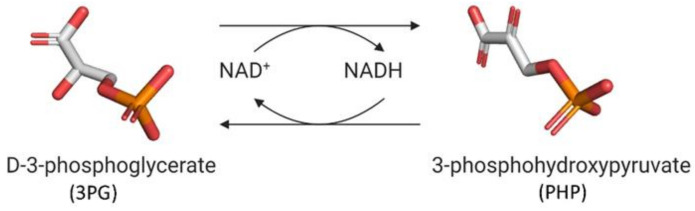
First reaction of the phosphorylated pathway: D-3-phosphoglycerate dehydrogenase catalyzes the NAD^+^-dependent conversion of D-3-phosphoglycerate into 3-phosphohydroxypyruvate.

D-3-phosphoglycerate dehydrogenase (PHGDH, EC 1.1.1.95) catalyzes the transformation of D-3-phosphoglycerate (3PG) into 3-phosphohydroxypyruvate (PHP) using NAD^+^/NADH as a cofactor (Figure 1). This is the first and the rate-determining step in the “phosphorylated pathway”, also termed the serine synthesis pathway [1]. The equilibrium of the reaction lies far in the direction of 3PG: at equilibrium, less than 5% of the substrate/product is in the form of PHP [2]; the reaction proceeds in the direction of serine synthesis because the product is continuously utilized in the subsequent steps. This provides a mechanism that preserves 3PG for later steps in glycolysis by using it in the phosphorylated pathway only when serine is required.

Serine supports a number of anabolic processes, including protein, lipid and nucleic acid synthesis. In humans, L-serine is available from different sources, i.e., from intestinal absorption of dietary proteins, degradation of proteins and phospholipids and conversion of glycine via serine hydroxymethyltransferase. The phosphorylated pathway represents the primary route of de novo L-serine synthesis, especially in the central nervous system [3], considering the low permeability of the amino acid at the blood–brain barrier [4]. This pathway involves three enzymes which are coordinately expressed in many tissues: phosphoserine aminotransferase (PSAT), phosphoserine phosphatase (PSP) and PHGDH. The key role of PHGDH in L-serine synthesis has been demonstrated through the generation of knock-out mouse models [3]. L-Serine is the precursor of D-serine, the coagonist of the N-methyl-D-aspartate receptors (NMDAR). NMDARs are critically involved in brain development and plasticity, cognitive functions and cellular excitotoxicity, and NMDAR dysfunction is often responsible for cognitive deficits that develop during normal aging and in neurological disorders [5,6]. D-serine is synthesized by serine racemase from L-serine and degraded by serine racemase and D-amino acid oxidase [7,8]: the control of D-serine synthesis by acting on human serine racemase or D-amino acid oxidase is a difficult task since both enzymes show a K_m_ for the D-amino acid that is 20-fold higher than physiological concentration [7,9,10]. For a comprehensive recent review on L-serine and neurotransmission, see [11].

The overexpression of the gene encoding PHGDH has been identified in human cancers, particularly in breast cancers and melanomas [12,13,14,15], and PHGDH inhibition was reported to reduce proliferation. PHGDH is involved in nucleotide synthesis by supporting central one-carbon metabolism [16]. Furthermore, in the last decade, several mutations have been correlated to PHGDH deficiency [17,18,19]. Five homozygous missense mutations have been identified in several individuals with congenital microcephaly, psychomotor retardation and variable presence of seizures [17,20,21,22].

PHGDH has been characterized in various organisms, from bacteria to mammals; for a review, see [23]. It is a cytosolic enzyme which exists in at least three different basic structural forms, named type I, II and III. Both *Mycobacterium tuberculosis* and human PHGDH (hPHGDH, made up of 533 amino acids for a mass of 56.8 kDa) belong to the structurally most complex type I (they share a 38% sequence identity). Actually, all types of PHGDH contain two common domains: the substrate-binding domain and the cofactor-binding domain; the type I members contain two additional regulatory domains at the C terminus, the ACT (aspartate kinase-chorismate mutase-tyrA prephenate dehydrogenase) and ASB (allosteric substrate-binding) domains. The NAD^+^ and the substrate-binding domains appear to be conserved among species, while sequence conservation is low or insignificant for the other two domains. In selected species, the ACT domain has been reported to function as a binding site for serine to provide feedback inhibition, although this regulatory mechanism was not evident for hPHGDH [24]. The structure of a truncated version of hPHGDH, named sPHGDH (residues 3-314), which contains the substrate-binding domain and the cofactor-binding domain only, was solved [24].

In recent years, various studies have been conducted on the full-length and the truncated versions of hPHGDH, such as the binding and kinetics of the physiological substrate 3PG and alternative substrates [24,25,26] and the identification of inhibitors [27,28]. Here, we performed an in-depth biochemical characterization of recombinant full-length hPHGDH. This represents an essential step in understanding the physiological production of L-serine through the phosphorylated pathway (and, in turn, of D-serine) and in identifying the alterations due to hPHGDH mutations that are related to pathological states.

## 2. Results

### 2.1. Expression of Recombinant hPHGDH

His-tagged full-length hPHGDH was expressed in BL21(DE3) *E. coli* cells carrying the pETM-hPHGDH expression plasmid. Following the addition of isopropyl-β-D-thiogalactoside (IPTG), the maximal activity value in the crude extract was observed after 20 h at 20 °C, corresponding to 0.12 units/mg of total proteins. The recombinant hPHGDH was purified from the crude extract by chromatography on a HiTrap Chelating column and was nearly homogeneous after this single step: SDS-PAGE analysis showed a single band of the expected size (~60 kDa vs. a theoretical value of 59.776 kDa, Figure S1). After dialysis, the preparation had a specific activity of 1.3 U/mg of protein. From 1 L of culture, 125 U (96 mg) of purified enzyme was obtained with an 83% recovery yield (Table S1). While a number of studies have focused on the truncated sPHGDH form (see Section 1), three papers reported on the expression of full-length hPHGDH in *E. coli* cells as the His-tagged form, although no details on the production levels were reported [24,27,29].

### 2.2. Kinetic Properties and Substrate Specificity

The reaction of recombinant hPHGDH on 3PG (forward direction) was assayed following the fluorescence signal related to the redox state of the nicotinamide cofactor and removing the PHP product using PSAT (the following enzyme in the phosphorylated pathway) or 200 mM hydrazine. The enzymatic activity of recombinant hPHGDH was similar in 50 mM HEPES at pH 7.5, 25 mM HEPES at pH 7.0, and 50 mM TrisHCl at pH 8.5 buffer (not shown), while it was strongly affected by phosphate (see below). When assayed using a multicomponent buffer, the recombinant hPHGDH showed the highest activity at pH ~8 and apparent pK_a_ values at 2.8, 7.4 and 10.2; see Figure S2A. Notably, the enzyme was stable after 30 min of incubation at 4, 25 and 37 °C and at pH values ≥4.0 and ≤9.0, while at longer incubation times, the activity was largely maintained in the pH range 4–5 only (see Figure S2B for the time course following the incubation at 37 °C). The highest residual activity was maintained at 4 °C, independently of the pH (not shown).

When the hPHGDH activity was measured in the physiological direction by the PSAT-coupled assay at 0.12 mM NAD^+^ and increasing 3PG concentrations (Figure S3A), a K_m_ value of 0.26 mM for 3PG and a k_cat_ of 1.5 s^−1^ were determined using a classical Michaelis–Menten equation: no evidence of allosteric behavior at low substrate concentration was apparent, and no substrate inhibition was observed at high 3PG concentration (up to 15 mM). Notably, very close kinetic parameters were determined using hydrazine to eliminate the reaction product instead of PSAT (Figure S3A and Table 1). Using a fixed and saturating amount of 3PG (i.e., 2.5 mM) and increasing NAD^+^ concentrations, a k_cat_ of 2.2 s^−1^ was determined (Figure S3A). This latter value is higher than the one determined at 120 μM NAD^+^; this apparent discrepancy is due to the use of a subsaturating NAD^+^ concentration in the previous reaction (the corresponding K_m_ value is 0.15 mM). Accordingly, the forward reactions were assayed at 1.5 mM NAD^+^ (Figure S3): the corresponding values are reported in Table 1.

To gain insights into the substrate preferences of hPHGDH, compounds similar to 3PG, such as D-3-hydroxybutyrate, D-lactate, D-tartrate and D-malate, were tested. All these compounds were oxidized by hPHGDH but with K_m_ values higher than the one for the physiological substrate 3PG (see Figure S3D–F and Table 1) and with a lower k_cat_ value for D-tartrate and D-malate (this pointing to differences in the microscopic rate constants limiting the turnover number). We conclude that these compounds are not likely to compete with 3PG for dehydrogenation by hPHGDH under physiological conditions.

When the hPHGDH reaction was assayed in the opposite direction at 150 μM NADH and increasing PHP concentrations, an apparent k_cat_ of 1.3 s^−1^ and a K_m_ of 14 μM for PHP were determined; a substrate inhibition effect was apparent at PHP concentrations higher than 0.3 mM (K_i_ = 2.23 mM, Figure S4 and Table 1), confirming the result reported by [26]. Using a fixed and saturating amount of PHP (0.5 mM), the recombinant enzyme displayed an apparent k_cat_ of 3.4 s^−1^ and a K_m_ for NADH of 0.56 mM (Figure S4B); accordingly, the reverse reactions were assayed using 5 mM NADH (Table 1). The reverse reaction was also assayed on α-ketoglutarate and oxaloacetate (Figure S4); for the former substrate, the kinetic parameters resembled those determined for PHP with a substrate inhibition effect at concentrations higher than 1 mM (K_i_ ~13 mM), while for oxaloacetate, a higher apparent K_m_ value was determined (see Table 1). Interestingly, from a kinetic point of view, α-ketoglutarate could compete with PHP for NADH reduction by hPHGDH in vivo, generating the oncometabolite D-2-hydroxyglutarate.

α-Ketoglutarate can be generated by the reaction of PSAT, the next enzyme in the phosphorylated pathway, using L-glutamate as an amino donor, as reported by [25]. As the equilibrium of the reaction catalyzed by hPHGDH is largely favored in the direction of 3PG formation (reverse reaction), only a small amount of NADH is generated under standard conditions in the presence of 2.4 mM 3PG and 0.12 mM NAD^+^. We observed that NADH production is faster when hPHGDH is coupled with PSAT in the presence of 2.5 mM 3PG, 0.12 mM NAD^+^ and 30 mM L-glutamate, which, instead, is reversed when adding 10 mM α-ketoglutarate and omitting PSAT. The observation that a specific reoxidation step of NADH is not required in the presence of PHGDH, PSAT and L-glutamate needs further investigation.

### 2.3. Cofactor Binding

**Figure 2:**
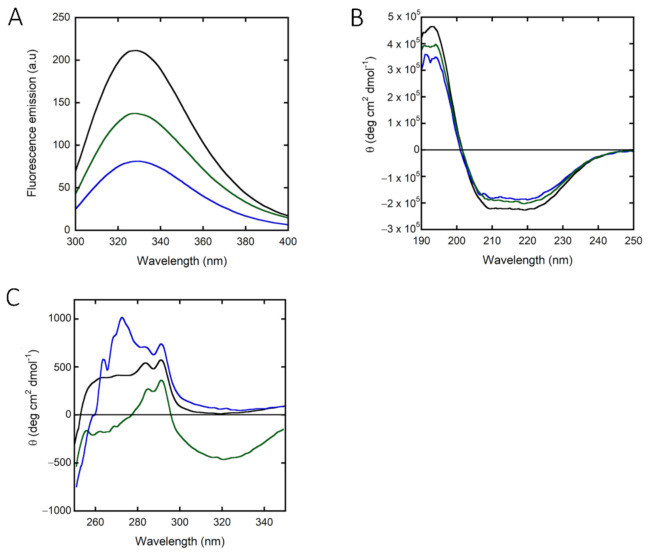
Spectral properties of recombinant hPHGDH alone (black) and in the presence of 0.12 mM NAD^+^ (blue) or 0.10 mM NADH (green). Comparison of: (**A**) fluorescence emission spectra (0.1 mg/mL, excitation at 280 nm); (**B**) far-UV CD spectra (0.1 mg/mL); (**C**) near-UV CD spectra (0.4 mg/mL).

The absorbance spectrum of hPHGDH shows a maximum intensity at 280 nm, and the protein fluorescence spectrum, following excitation at 280 nm, shows a peak at 330 nm. Interestingly, the fluorescence intensity is quenched by adding NADH and, most significantly, NAD^+^ (Figure 2A), indicating that cofactor binding induced a conformational change. 

The far-UV CD spectrum of hPHGDH, with minima at 208 and 222 nm (Figure 2B), is indicative of a large amount of α-helices (approx. 62% vs. 5% of β-sheets, as calculated using the DichroWeb software, Selcon3 method) [30]. The cofactor slightly altered the secondary structure content: in the presence of NAD^+^ or NADH, the percentage of α-helices decreased to 55% and 52%, respectively, while the value corresponding to β-sheets content increased slightly (9% and 11%, respectively) (Figure 2B). Concerning the near-UV CD spectrum, the addition of both redox forms of the nicotinamide cofactor significantly altered the 250 to 290 nm region, corresponding to the signal for aromatic residues (Figure 2C), pointing to a protein conformational change associated with cofactor binding. In detail, a different contribution of the signal due to tyrosine residues (at ~280 nm) was observed following NAD^+^ binding (increase) vs. NADH binding (decrease); in both cases, an alteration of the tryptophan region of influence (at ~290 nm) was also apparent.

**Figure 3:**
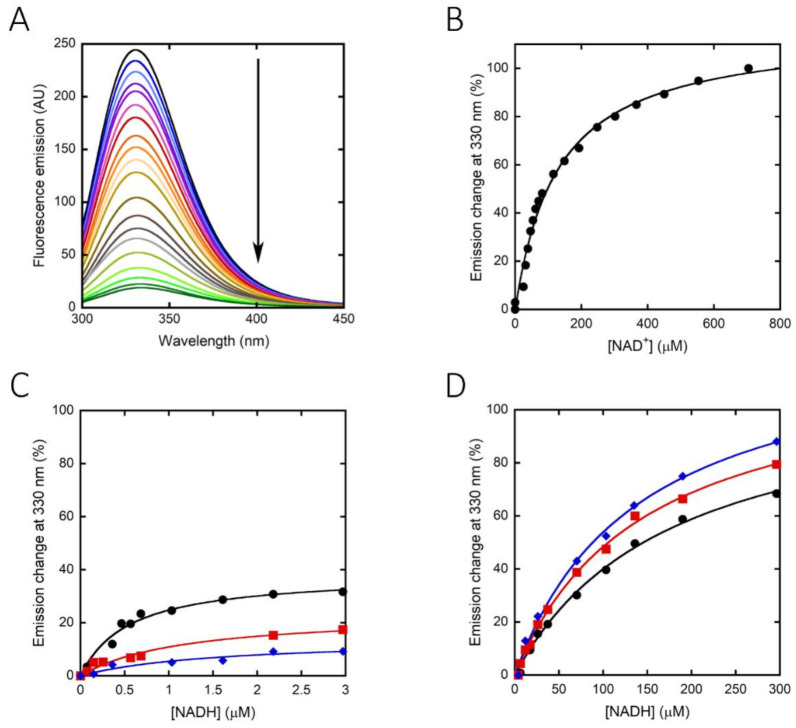
Effect of cofactor binding on the protein fluorescence emission spectrum of hPHGDH (1 μM): NAD^+^ (**A**,**B**) and NADH (**C**,**D**). Arrow in panel A indicates the spectral changes at increasing NAD^+^ concentration. In panels C and D, the binding of NADH was evaluated in the absence (black) and presence of 0.25 (red) or 2.5 mM (blue) 3PG. Panel C shows the fit of the values, expressed as a percentage of the total fluorescence change, corresponding to the first saturation phase of the protein fluorescence change (up to 3 μM of cofactor concentration). Panel D shows the best fit for the second phase of saturation. Measurements were performed at 15 °C and repeated three times for each condition.

The binding constant for the nicotinamide cofactor was determined by titrating the enzyme with increasing amounts of NAD^+^ or NADH and monitoring holoenzyme reconstitution, following the fluorescence intensity at 330 nm (using excitation wavelengths at both 280 and 298 nm). The binding of NAD^+^ to hPHGDH was a monophasic process, with a K_d_ value of 130 µM (Figure 3A,B), while the quenching in protein fluorescence intensity vs. NADH concentration was biphasic (Figure 3C,D); a first saturation was apparent up to ∼3 μM NADH, corresponding to ∼35% of the change in fluorescence intensity, and a second saturation was evident up to ∼300 μM NADH. The corresponding K_d_ values are 0.49 and 168 µM, respectively (Table 2). The binding of NADH to hPHGDH was also investigated in the presence of an amount of 3PG corresponding to its K_m_ value (0.25 mM) or to a saturating amount (2.5 mM): the change in fluorescence intensity was again biphasic, but the amplitude of the first phase decreased to 20% and 12% of the total change, respectively. K_d_ values for the first phase were 1.2 and 1.7 μM at 0.25 and 2.5 mM 3PG, respectively, while those for the second phase (∼130 μM) were similar to the value determined for the free enzyme (Table 2). These results suggest an equilibrium between two hPHGDH forms differing in NADH affinity; the presence of a ligand in the active site favors the species at lower affinity.

The effect of temperature on protein unfolding was evaluated by monitoring spectral signals related to the protein tertiary structure. Following the changes in protein fluorescence intensity at 330 nm (due to both Trp and Tyr residues) [31], a melting temperature (T_m_) of 48.4 °C was determined for the enzyme alone, a figure that increased to 52.4 and 55.7 °C for the enzyme incubated with 120 µM NAD^+^ or 100 µM NADH (Table 3). Slightly different values were determined by following the circular dichroism signal at 280 nm (characteristic of Tyr residues) [32]; T_m_ was 47.5 °C for the protein alone and 48.2 and 51.0 °C for the protein with 120 µM NAD^+^ or 100 µM NADH, respectively. This result suggests that cofactor binding, especially NADH, alters hPHGDH conformation, driving the acquisition of a more stable structure.

### 2.4. Ligand Binding

hPHGDH activity was affected by the presence of mono- or divalent ions (K^+^, Na^+^, Ca^2+^ and Mg^2+^); the strongest effect was a 40% decrease at 10 mM KCl (Figure S5). The latter influence might be of physiological relevance, since the KCl level in cytosol is ~160 mM. The activity of hPHGDH on 2.4 mM 3PG was not affected by the product PHP (up to 50 mM final concentration) and only marginally affected by the presence of D-serine, L-serine and glycine; the most evident effect was a ~25% decrease at 250 mM D-serine (a nonphysiological concentration; see Figure S6).

**Figure 4:**
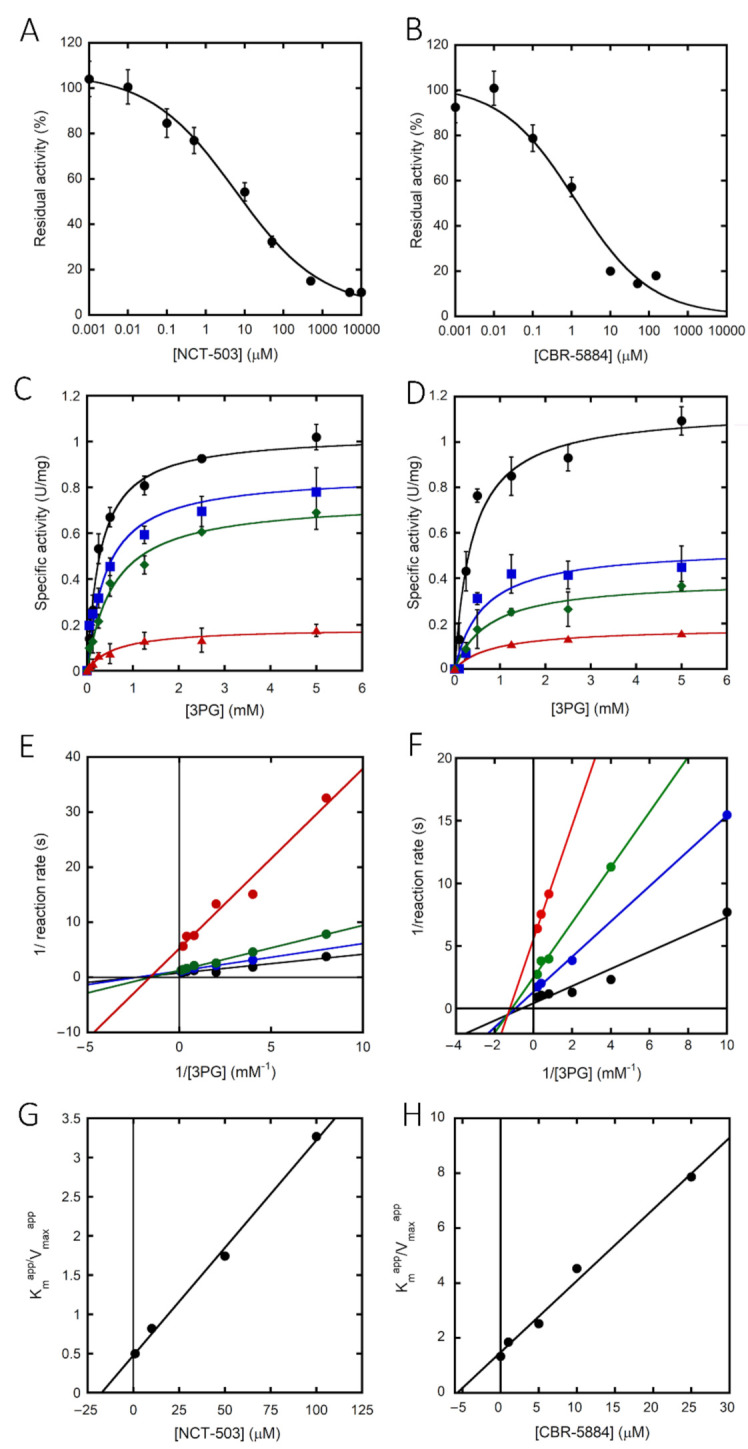
Inhibition of hPHGDH activity. hPHGDH inhibition assays performed on 3PG and NAD^+^ (at fixed concentration of 2.4 and 0.12 mM, respectively) in the presence of different amounts of NCT-503 (**A**) or CBR-5884 (**B**). (**C**,**D**) Kinetics of hPHGDH on 3PG in the presence of increasing concentrations of NCT-503 (at 0, 1, 10 and 100 µM; lines from top to bottom) or CBR-5884 (at 0, 0.1, 1 and 10 µM; lines from top to bottom). Data are the average of three determinations and error bars represent mean ± SD. (**E**,**F**) Double reciprocal plot of data as in panels C and D. K_i_ value for both the inhibitors was estimated by fitting the apparent K_m_/V_max_ values calculated at different inhibitor concentrations (**G**,**H**).

In recent years, several investigations have focused on identifying and characterizing new inhibitors of hPHGDH for their potential use as drugs to treat cancer progression [27,28,33]. Here, we selected two of these molecules and evaluated their effect on full-length hPHGDH. NCT-503 inhibited enzymatic activity, yielding an IC_50_ of 5.8 μM (Figure 4A and Table 2). The kinetics of hPHGDH on 3PG were thus investigated at increasing NCT-503 concentration; as shown by the plots reported in Figure 4C,E, the inhibitor was able to alter both the apparent maximal rate and the K_m_ value for 3PG. Thus, NCT-503 acts as a noncompetitive inhibitor; a K_i_ of 23.4 µM was estimated (see Figure 4G). Even the CBR-5884 compound affected hPHGDH activity; the IC_50_ was ~1.0 µM (Figure 4B). A noncompetitive inhibition was also apparent for CBR-5884 from the plots reported in Figure 4D,F; a K_i_ ~5.7 µM was estimated (see Figure 4H and Table 2).

We then investigated the ability of the two inhibitors to alter hPHGDH conformation by following the quenching of protein fluorescence. A decrease in protein fluorescence was apparent at increasing concentrations of NCT-503, yielding a K_d_ value of 24 μM (similar to the K_i_ value; Table 2). The same analysis was not feasible for CBR-5884 due to the strong fluorescence observed at >150 µM concentration.

### 2.5. Oligomeric State and Phosphate Effect

On gel permeation chromatography using a Superdex 200 Increase column, the recombinant hPHGDH eluted as a major peak corresponding to approx. 240 kDa (Figure S7A), indicating that it behaved as a tetramer. A second minor peak at ~480 kDa was apparent at increasing concentrations of potassium phosphate, as confirmed by Western blot analysis, indicating that an octameric oligomer representing 9–20% of the total protein was present. This seems to be a specific effect due to phosphates as the peak corresponding to the octameric form was absent in 20 mM phosphate buffer and was not generated when the ionic strength was increased by adding NaCl (Figure S7A). Notably, a similar effect was apparent for *M. tuberculosis* PHGDH [34].

The enzymatic activity is strongly affected by phosphate ions; 30% of the activity was lost at 1 mM potassium phosphate (corresponding to the cellular level), and it was fully abrogated at 400 mM phosphate (Figure S7B, left). The time course of the activity assay showed that the inhibition occurred quickly after adding 250 mM potassium phosphate (see Figure S7B, right). Interestingly, the reverse reaction was similarly affected by the potassium phosphate concentration (compare blue and red bars in Figure S7B). When KCl or NaCl was used instead of potassium phosphate, 30% of the initial activity was also lost at ≥10 and >20 mM salt concentration, respectively, and the activity was never fully abolished (Figure S8). Indeed, a similar behavior was apparent at increasing concentrations of sodium tartrate, a compound used to generate crystals of truncated sPHGDH [24].

### 2.6. Conformational Changes

The binding of NADH and the effect of salt concentration (see above) as well as the ability of tartrate to generate crystals of truncated sPHGDH suggest that different conformational states exist in solution of this complex enzyme. We used limited proteolysis to evaluate the presence of such hPHGDH species based on the assumption that specific regions of the protein are differently susceptible to trypsinolysis in the free and complexed forms. Following the addition of 5% (*w*/*w*) trypsin, the full-length 56 kDa form of hPHGDH was quickly converted (in 120 min) into a ~40 kDa band, with the transient presence of a ~47 kDa species and additional products at 28, 25, 15 and 10 kDa (Figure S9A). The band at ~40 kDa should be expected to arise after deleting the C-terminal portion, thus lacking the ASB and ACT domains (residues 1–370). Based on a model of hPHGDH structure, we predict Arg370 as a susceptible site to trypsinolysis since it is located in a linker region connecting the substrate-binding domain and the cofactor-binding domain with the two additional regulatory domains ACT and ASB. Proteolysis of the full-length enzyme was hampered in the presence of 250 mM potassium phosphate or 500 mM sodium tartrate (Figure S9B,C) and, to a lesser extent, by 0.1 mM NCT-503 (Figure S9D). On the other hand, the generation of the 40 kDa species was favored by adding the cofactor NAD^+^, the substrate 3PG or both (Figure S9E–G).

## 3. Discussion

In this paper, we report on the recombinant expression of full-length hPHGDH in *E. coli* cells. By optimizing the growth conditions, ~100 mg of pure enzyme per liter of fermentation broth was obtained. Recombinant hPHGDH was easily purified by a single chromatographic step, and this allowed us to biochemically characterize the enzyme.

The full-length hPHGDH showed the highest activity at pH 8, similarly to the *E. coli* counterpart, a behavior that distinguishes it from *M. tuberculosis* PHGDH, which displayed optimal activity at pH 6.5 [23]. The hPHGDH activity was not affected by the buffer composition (with the only exception of phosphate; see below), and the enzyme showed an appreciable thermostability for a mesophilic protein (T_m_ ~48 °C). The binding of NAD^+^ to hPHGDH is a monophasic process, while the binding of NADH is biphasic; similar K_d_ values were apparent for NAD^+^ and for the main phase of NADH binding (~0.13 mM; Table 2), while the minor phase of NADH binding was characterized by a higher affinity. The amplitude of the minor phase decreased at increasing 3PG concentrations, pointing to the presence of two protein conformations in solution whose equilibrium is affected by the active-site ligand. Such conformations might be related to the asymmetrical tetramer made up of independent dimers used to explain the flip-flop reaction mechanism of *E. coli* PHGDH [35]. Notably, similar K_m_ values were determined for the oxidized and reduced forms of the nicotinamide cofactor when the forward and reverse reactions were studied (0.15 mM for NAD^+^ vs. 0.56 mM for NADH). Previous work on the truncated hPHGDH form has reported a lower K_d_ value for NADH binding based on isothermal calorimetry analyses (=0.2 µM, resembling the value for the first phase we observed; see Table 1) [24], while the value for NAD^+^ binding corresponded to the value we determined for the full-length enzyme; apart from the different protein and method used, no other explanation can be provided to justify such a difference. The cofactor binding affected the tertiary structure of hPHGDH and increased its thermostability (especially NADH; see Table 3), as previously reported [24].

hPHGDH catalyzed a reversible reaction (Figure 1); the reverse reaction starting from PHP was strongly favored. We investigated the forward reaction using two coupled assays and defined the experimental conditions (25 mM Hepes buffer at pH 7 and 37 °C). Notably, the kinetic parameters determined using the coupled assay with PSAT overlapped with those determined with hydrazine (Figure S3A), suggesting the latter is a simple and suitable method to assay the hPHGDH forward reaction. 

hPHGDH is a promiscuous enzyme, active on various carboxylic acids containing 3 to 5 carbon atoms (Table 1). 3PG is the best substrate due to its low apparent K_m_ (0.36 mM at 1.5 mM NAD^+^); all the alternative substrates showed K_m_ values of orders of magnitude higher, thus likely not affecting the reaction of hPHGDH on 3PG at the physiological level. The K_m_ values previously reported for hPHGDH (i.e., 0.18, 0.26 and 0.48 mM) [24,25,35] are in good agreement with the value we determined. In any case, discrepancies are also apparent; in a previous work, the kinetics with 3PG showed a negative cooperativity (Hill coefficient n = 0.63–0.67), and α-ketoglutarate was not efficiently utilized to produce α-hydroxyglutarate [35]. On the other hand, when a commercial preparation of hPHGDH was used [25], no cooperativity with 3PG was described (as well as in [24]), and similar k_cat_ values for 3PG and α-ketoglutarate were determined, as in our analyses (Table 1).

Concerning the reverse reaction, an apparent k_cat_ value similar to that for the forward reaction was determined (Table 1), but, since the K_m_ for PHP was ~10 µM (vs. a figure of 85 and 40 µM for the *M. tuberculosis* and the *E. coli* enzymes) [23,36], the kinetic efficiency k_cat_/K_m_ ratio was 20-fold higher than for the forward reaction on 3PG. Such a value for hPHGDH is lower than those reported for the *M. tuberculosis* and the *E. coli* enzymes, corresponding to 5.6 × 10^6^ M^−1^s^−1^ and 7 × 10^5^ M^−1^s^−1^, respectively [23,36]. hPHGDH also efficiently uses α-ketoglutarate as substrate, analogously to the enzyme from *E. coli* (showing a comparatively higher K_m_ and lower kinetic efficiency, i.e., 0.5 mM and 3 × 10^4^ M^−1^s^−1^) and differently from the ones from *M. tuberculosis* and rat liver [23]. The observed reduction of α-ketoglutarate by hPHGDH generating the oncometabolite D-2-hydroxyglutarate confirms a study based on commercial enzyme preparation [25], although the previous study reported a very slow reaction rate and a K_m_ of ~10 mM for α-ketoglutarate. Nevertheless, in both investigations, the k_cat_ values for 3PG and α-ketoglutarate were very close (see Figure S2A,C in [25]), and the specific activity on 3PG of the commercial enzyme used in [25] was in good agreement with the value for our hPHGDH preparation (~1.3 U/mg protein). Indeed, we suggest that the use of α-ketoglutarate (generated by PSAT from L-glutamate or by the cellular metabolism) by hPHGDH provides for a mechanism in which L-serine production could proceed without a net consumption of NAD^+^. Interestingly, for the reverse reaction on both PHP and α-ketoglutarate, a substrate inhibition effect was apparent (K_i_ in the millimolar range), confirming previous results on human, *M. tuberculosis* and rat liver PHGDHs [26,37].

hPHGDH activity is partially affected by mono- and divalent ions (Figure S5) and not affected by relevant amino acids linked to the phosphorylated pathway, such as D- and L-serine and glycine (Figure S6). The absence of L-serine inhibition indicates that no feedback inhibition is active for hPHGDH, in contrast to that of the *E. coli* and the *M. tuberculosis* counterparts [35,37].

The binding of two known inhibitors was studied on the full-length enzyme; both NCT-503 and CBR-5884 compounds inhibited the forward reaction of hPHGDH by a noncompetitive inhibition mechanism and one- or two-digit micromolar dissociation and inhibition constants (Table 2). The results for CBR-5884 do not differ significantly from those determined using the truncated sPHGDH form [38].

hPHGDH in solution is a homotetramer of ~240 kDa, like the enzymes from *E. coli* [39], rabbit liver [40] and chicken liver [41]. In the presence of phosphate ions, a second minor peak was generated corresponding to a molecular mass of ~480 kDa, i.e., an octameric oligomer. Such an effect was not due to the ionic strength of the solution, as the minor peak was not generated at increasing KCl concentrations. The presence of phosphate strongly and quickly inhibited hPHGDH (Figure S7); 30% of the activity was lost at 1 mM phosphate, a physiological cellular concentration. A conformational change induced by phosphate was proposed for *M. tuberculosis* PHGDH, suggesting that it affects the interaction between the ACT and the ASB domains compared to the structure solved in the presence of 1 M tartrate [34]. Awaiting the resolution of the 3D structure of free and complexed full-length hPHGDH, we used different techniques to highlight changes in protein conformation, such as spectral analyses (Figure 2), temperature-induced unfolding (Table 3) and limited proteolysis. The latter experiments suggest that the C-terminal portion, harboring the allosteric ACT and ASB domains, can be easily cleaved by trypsin and that phosphate and tartrate protect hPHGDH from this cleavage (Figure S9). Indeed, NAD^+^ and 3PG protect the ~40 kDa protein species from further proteolysis. Similarly to that observed at increasing phosphate concentrations, NaCl, KCl and sodium tartrate also inhibited hPHGDH (Figure S8). Taken together, several lines of evidence suggest that different conformations and/or oligomeric states exist for hPHGDH.

The nonessential amino acid L-serine is critical for a plethora of metabolic pathways and physiological processes; thus, a deeper knowledge of its biosynthetic pathway is essential to shed light on related defects. hPHGDH is a complex enzyme made by four domains, possessing an oligomeric organization, catalyzing a reversible reaction and showing substrate and catalytic promiscuity; by investigating this enzyme, we can identify novel approaches to reduce cancer progression and to modulate neurotransmission in disorders due to L-serine deficiency and to NMDAR-altered functioning.

## 4. Materials and Methods

### 4.1. Design, Synthesis and Cloning of hPHGDH Gene

The synthetic gene encoding hPHGDH was designed by in silico back translation of the amino acid sequence reported in the data bank (GenBank accession number CAG33076.1). The codon usage was optimized for expression in *E. coli*. The gene was produced by Invitrogen (*hPHGDH*_pMA-T). The cDNA molecule was cloned into the pETM11 expression plasmid with *Nco*I and *Xho*I (Roche, Milan, Italy) restriction sites (pETM-hPHGDH plasmid). With this cloning strategy, a 6xHis-tag encoded by the plasmid was added at the N-terminus of the protein that could eventually be eliminated by cleavage with TEV protease. The cloned regions were confirmed by sequencing (Euroclone, Milan, Italy).

### 4.2. Expression and Purification of Recombinant hPHGDH and hPSAT

For hPHGDH overexpression in *E. coli*, BL21(DE3) cells (Invitrogen, Milan, Italy) transformed with the pETM-hPHGDH expression plasmid were aerobically grown at 37 °C in Luria Bertani broth up to OD_600nm_ ≈ 0.6–0.8. The growth was then stopped by incubation for 30 min on ice before adding 0.5 mM IPTG to induce the expression of the enzyme. Growth was continued for another 20 h at 20 °C. Cells were harvested by centrifugation (6000× *g*, 30 min, 4 °C) and frozen. Pellets were resuspended in lysis buffer (50 mM potassium phosphate, 10 mM MgCl_2_, 200 mM KCl, 10% (*v*/*v*) glycerol, 1 µg/mL DNAse, 0.7 µg/mL pepstatin and 0.19 mg/mL PMSF) using 3.5 mL/g cells; the cells were disrupted by sonication for 2–3 min. Cell debris was removed by centrifugation at 27,000× *g* for 1 h and the crude extract purified by chromatography on a HiTrap Chelating column (Invitrogen, Milan, Italy) loaded with NiCl_2_ and using the following solutions: starting buffer, 50 mM sodium pyrophosphate, pH 7.5, 300 mM NaCl and 2% (*v*/*v*) glycerol; elution buffer, 50 mM sodium pyrophosphate, pH 7.5, 0.5 M imidazole and 2% (*v*/*v*) glycerol. The final enzyme preparations were stored at −80 °C in 50 mM sodium pyrophosphate, pH 7.5, 10 mM MgCl_2_, 25 mM NaCl, 2% (*v*/*v*) glycerol and 2.5 mM 2-mercaptoethanol (added for long-term storage). The enzyme concentration was determined spectrophotometrically by using the theoretical extinction coefficient at 280 nm (40.45 mM^−1^ cm^−1^). PSAT was expressed in *E. coli* BL21-CodonPlus^®^-RIL cells and purified as detailed elsewhere [42]. Briefly, bacteria were grown in M9-glucose medium containing 50 μg/mL kanamycin and 34 μg/mL chloramphenicol at 37 °C and transferred, after induction with 1 mM IPTG, at 4 °C and grown for 3 days. The protein was purified by IMAC on a Talon cobalt affinity resin using an AKTA apparatus. The final protein preparation was stored in 25 mM Tris, 300 mM NaCl, 4 μM PLP and 1 mM TCEP, pH 8.0, at −80 °C. The final PSAT preparation had a specific activity of about 10 U/mg protein.

### 4.3. Activity Assay and Kinetic Measurements

hPHGDH activity in the physiological direction and the apparent kinetic parameters on 3PG were determined by monitoring NADH fluorescence (recording the emission at 450 nm following excitation at 360 nm) over time at 37 °C in 96-well plates with TECAN Infinite 200 Pro (Männedorf, Switzerland), a procedure partially modified from [28]. PSAT was added to drive the reaction and to avoid product inhibition of hPHGDH. One unit corresponds to the amount of enzyme that converts 1 µmol of 3PG in one minute at 37 °C. The assays were performed using 0.04 µg hPHGDH (0.001 U) in 25 mM Hepes, pH 7.0, on 2.5 mM 3PG and in the presence of 120 µM or 1.5 mM NAD^+^, 30 mM glutamate and 0.032 U of PSAT.

The apparent kinetic parameters on 3PG and NAD^+^ were determined using both the PSAT-coupled assay (in the presence of 2.5, and 0.120 or 1.5 mM of the substrate or cofactor, respectively) and the procedure from [25], in which 200 mM hydrazine was added instead of PSAT to drive the reaction forward. The latter assay was used to determine the apparent kinetic parameters on alternative substrates. K_m_ and V_max_ values were calculated according to a Michaelis–Menten equation using the initial velocity values determined at increasing substrate concentrations, i.e., up to 15 mM 3PG, 10 mM NAD^+^, 500 mM D-malic acid, 500 mM 3-hydroxybutyric acid, 500 mM D-tartaric acid and 300 mM D-lactic acid. The kinetics on 3PG were determined with both assays. hPHGDH activity in the reverse direction was also assayed monitoring the fluorescence change at 450 nm, using increasing substrate concentration and 150 μM or 5 mM NADH. Plots of activity vs. substrate concentration were fit to the general Michaelis–Menten equation modified to account for a substrate inhibition effect yielding complete inhibition and no cooperativity [26]: v = V_max_ [S]/[1 + (K_m_ + [S] + ([S]^2^/K_i_)]. The k_cat_ values were calculated per monomer (of 59.8 kDa). The concentration of (intact) PHP and α-ketoglutarate solutions was confirmed by measuring the level of NADH conversion using an excess of hPHGDH and bovine L-glutamate dehydrogenase, respectively.

The effects of KCl, NaCl, MgCl_2_ and CaCl_2_ and of pH and temperature on enzyme activity and stability were investigated using the hydrazine assay. The activity was assayed after incubation at 4, 25 or 37 °C of 1.4 mg/mL hPHGDH at different pH values in a multicomponent buffer (15 mM Tris-HCl, 15 mM Na_2_CO_3_, 15 mM H_3_PO_4_, 100 mM KCl and 1% (*v*/*v*) glycerol) for up to 1400 min [43]. The dependence of activity on pH was fitted using an equation for three pK_a_ values, according to [44], providing apparent values unrelated to microscopic ones.

The hydrazine assay was used to evaluate the effect of phosphate and KCl, serine synthesis metabolites (D-Ser, L-Ser and Gly) and inhibitors (NCT-503 and CBR-5884 dissolved in DMSO; the maximal solubility of the CBR-5884 is ≤1 mM) on hPHGDH activity, and thus to determine IC_50_ and K_i_ values and the mechanism of inhibition.

hPHGDH activity in the reverse direction was determined by monitoring the decrease in NADH fluorescence over time at 37 °C. The assays were performed using 0.02 U (0.6 µg) of enzyme in 25 mM Hepes, pH 7.0, on different amounts of PHP and in the presence of 0.15 mM NADH or on 0.5 mM PHP and different amounts of the cofactor.

### 4.4. Determination of Oligomerization State

Size-exclusion chromatography was carried out using a Superdex 200 Increase column on an AKTA system. The column was equilibrated with increasing concentrations (20, 100, 250 and 500 mM) of potassium phosphate, pH 7.0; 0.15 M NaCl was added at low phosphate concentrations (20 and 100 mM). The presence of hPHGDH in the eluted peaks was confirmed by SDS–PAGE and Western blot analyses by using a specific anti-His antibody (diluted 1:200, Santa Cruz Biotechnology Inc., Dallas, TX, USA).

### 4.5. Spectral Measurements

Circular dichroism (CD) spectra were recorded using a Jasco J-815 spectropolarimeter (Jasco Co., Cremella, Italy) in 10 mM potassium phosphate, pH 7.0, 15 °C. The cell pathlength was 0.1 cm for measurements in the 200 to 250 nm region (0.1 mg protein/mL) and 1 cm for measurements in the 250 to 350 nm range (0.5 mg protein/mL) [32].

Protein fluorescence spectra were measured at 0.06–0.1 mg/mL protein concentration in 10 mM potassium phosphate, pH 7.0, 15 °C; spectra were recorded using a Jasco FP-750 instrument and were corrected for the buffer contribution. Protein fluorescence spectra were recorded between 300 and 450 nm, with excitation at 280 and 298 nm.

The ligand dissociation constants (K_d_) were estimated by titrating 1 μM enzyme with increasing amounts of NAD^+^, NADH, NCT-503 or CBR-5884 and following the protein fluorescence quenching at 330 nm. All recorded spectra were subtracted for the contribution of the ligand (added to the buffer only) and were corrected for dilution at every addition. The K_d_ values for NADH to the hPHGDH moiety were determined in the presence and in the absence of 3PG (0.25 or 2.5 mM). In all cases, K_d_ values were determined by fitting the data to a hyperbolic function. Temperature-ramp experiments were performed in 10 mM potassium phosphate, pH 7.0. For this, a software-driven, Peltier-equipped fluorimeter was used to measure the protein fluorescence change at 330 nm or a CD spectropolarimeter to follow the CD signal at 280 nm in order to reproduce a temperature gradient (0.5 °C/min) [31].

### 4.6. Limited Proteolysis

hPHGDH at 0.4 mg/mL was incubated at 25 °C in 50 mM Hepes pH 7.5, 100 mM NaCl, 10% (*v*/*v*) glycerol, 1 mM EDTA and 5 mM 2-mercaptoethanol in the presence of 5% (*w*/*w*) trypsin (Roche, Basel, Switzerland). The cofactor NAD^+^ and the substrate 3PG, alone and together (0.12 and 2.4 mM, respectively), potassium phosphate (250 mM), D,L-tartaric acid (500 mM) and the inhibitor NCT-503 (0.1 mM) were added to the incubation mixture. At different times (0, 30, 60, 120 and 240 min), 8 µg of protein was diluted in SDS-sample buffer, heated at 95 °C for 5 min and analyzed by SDS-PAGE.

## Figures and Tables

**Table 1:** Apparent kinetic parameters of recombinant hPHGDH at 37 °C and pH 7.0. Values are per monomer.

Substrate	Formula	k_cat_ (s^−1^)	K_m_ (mM)	k_cat_/K_m_ (mM^−1^s^−1^)	Assay ^a^	Conditions ^b^
**Forward reaction:**						
3-Phosphoglycerate (3PG)	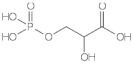	1.48 ± 0.37	0.255 ± 0.059	5.80	1,2	1a
		2.97 ± 0.09	0.360 ± 0.061	8.25	2	1b
D-Lactate	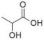	5.41 ± 0.15	151 ± 9	0.036	2	1b
D-3-Hydroxybutyrate	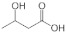	2.53 ± 0.27	41 ± 19	0.061	2	1b
D-Tartrate	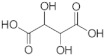	0.41 ± 0.03	5.55 ± 2.73	0.074	2	1b
D-Malate	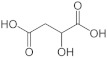	0.44 ± 0.01	4.48 ± 0.87	0.098	2	1b
NAD^+^	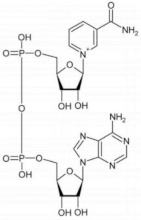	2.23 ± 0.26	0.148 ± 0.021	15.1	1,2	2
**Reverse reaction:**						
3-Phosphohydroxypyruvate (PHP)	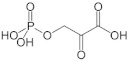	1.29 ± 0.09	0.014 ± 0.003 [K_i_ = 2.23 ± 0.37]	91.1	3	3a
		2.28 ± 0.14	0.010 ± 0.003 [K_i_ = 1.26 ± 0.25]	228		3b
α-Ketoglutarate	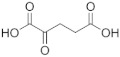	1.03 ± 0.10	0.024 ± 0.007 [K_i_ = 13.3 ± 4.6]	39.1	3	3b
Oxaloacetate	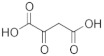	1.76 ± 0.20	0.90 ± 0.42 [K_i_ = 152 ± 56]	1.96	3	3b
NADH	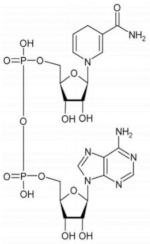	3.37 ± 0.12	0.559 ± 0.075	6.0	3	4

^a^ The forward reaction was assayed using a coupled assay with PSAT (1) or by reacting the product PHP with 200 mM hydrazine (2). The reverse reaction was assayed following the redox state of the cofactor as fluorescence change at 450 nm (3). ^b^ The assay was performed at 37 °C in 25 mM Hepes pH 7.0 and (1) a: 120 µM NAD^+^, b: 1.5 mM NAD^+^; (2) 2.5 mM 3PG; (3) a: 150 µM NADH, b: 5 mM NADH; (4) 0.5 mM PHP.

**Table 2:** Binding of nicotinamide cofactors and selected inhibitors to hPHGDH.

Ligand	K_d_ (µM)	Assay
NAD^+^	130.3 ± 8.3	Quenching of protein fluorescence (at 15 °C)
NADH ^a^ first phase (free)	0.49 ± 0.03 (35%)	Quenching of protein fluorescence (at 15 °C)
(+0.25 mM 3PG)	1.20 ± 0.02 (20%)	
(+2.5 mM 3PG)	1.71 ± 0.07 (12%)	
second phase (free)	169 ± 27 (65%)	
(+0.25 mM 3PG)	129 ± 23 (80%)	
(+2.5 mM 3PG)	134 ± 9 (88%)	
NCT-503	24.0 ± 5.0	Quenching of protein fluorescence (at 15 °C)
	IC_50_ = 5.75 ± 2.33	Inhibition of activity at 2.4 mM 3PG and 0.12 mM NAD^+^ (at 37 °C)
	K_i_ = 17.7	Inhibition of activity at different 3PG concentrations and 0.12 mM NAD^+^ (at 37 °C)
CBR-5884	IC_50_ = 1.02 ± 0.37	Inhibition of activity at 2.4 mM 3PG and 0.12 mM NAD^+^ (at 37 °C)
	K_i_ = 5.7	Inhibition of activity at different 3PG concentrations and 0.12 mM NAD^+^ (at 37 °C)

Reported values are mean ± SD for three measurements. ^a^ In parenthesis is reported the amplitude of protein fluorescence quenching associated with each phase.

**Table 3:** Melting temperature values determined for recombinant hPHGDH with or without the cofactors.

	T_m_ (°C)	
	CD (220 nm)	Fluorescence (330 nm)
hPHGDH	47.2 ± 0.5	48.4 ± 2.1
+120 μM NAD^+^	48.2 ± 1.7	52.4 ± 0.4
+100 μM NADH	51.0 ± 0.3	55.7 ± 0.1

## Data Availability

Data are contained within the article or supplementary material. The data presented in this study are available on request from the corresponding author.

## Supplementary Materials

The following materials are available online at https://www.mdpi.com/article/10.3390/ijms22084231/s1, Figure S1: Purification of hPHGDH from *E. coli* crude extract; Figure S2: Effect of pH on activity and stability of hPHGDH; Figure S3: Kinetics of hPHGDH in the forward direction on different substrates; Figure S4: Kinetics of hPHGDH in the reverse direction on different substrates; Figure S5: Effect of monovalent and divalent ions on hPHGDH activity at 2.4 mM 3PG and 0.12 mM NAD+; Figure S6: D-serine, L-serine and glycine only marginally affect the activity of hPHGDH; Figure S7: Effect of phosphate ions on hPHGDH properties; Figure S8: Effect of concentration of different salts on hPHGDH activity; Figure S9: Limited proteolysis of hPHGDH evaluated by SDS-PAGE; Table S1: Purification of recombinant hPHGDH.
