# Supplementary material for: Biochemical and Biophysical Characterization of Recombinant Human 3-Phosphoglycerate Dehydrogenase
Source: Int J Mol Sci. 2021 Apr 19;22(8):4231. doi: 10.3390/ijms22084231 (PMC8073719; doi:10.3390/ijms22084231)
Supplement: Supplementary file 1 [file ijms-22-04231-s001.zip › ijms-1142475-supplementary.pdf]

Supplementary

Biochemical and biophysical characterization of recombinant human 3-phosphoglycerate dehydrogenase

Giulia Murtas[1*], Giorgia Letizia Marcone[1], Alessio Peracchi[2], Erika Zangelmi[2], Loredano Pollegioni[1]

[1]Department of Biotechnology and Life Sciences, University of Insubria, via J.H. Dunant 3, 21100 Varese, Italy

[2] Department of Chemistry, Life Sciences and Environmental Sustainability, University of Parma, 43124 Parma, Italy

*Corresponding author: Giulia Murtas, Department of Biotechnology and Life Sciences, University of Insubria, via J.H. Dunant 3, 21100 Varese, Italy

e-mail address: g.murtas@uninsubria.it

Table S1. Purification of recombinant hPHGDH from 16 g of *E. coli* BL21(DE3) cells (2.8 L of fermentation broth).

Sample	Activity (U)	Proteins (mg)	Specific activity (U/mg)	Yield (%)	Purification index
Crude extract	363	2950	0.12	100	1
Pure enzyme	350	270	1.32	83	11

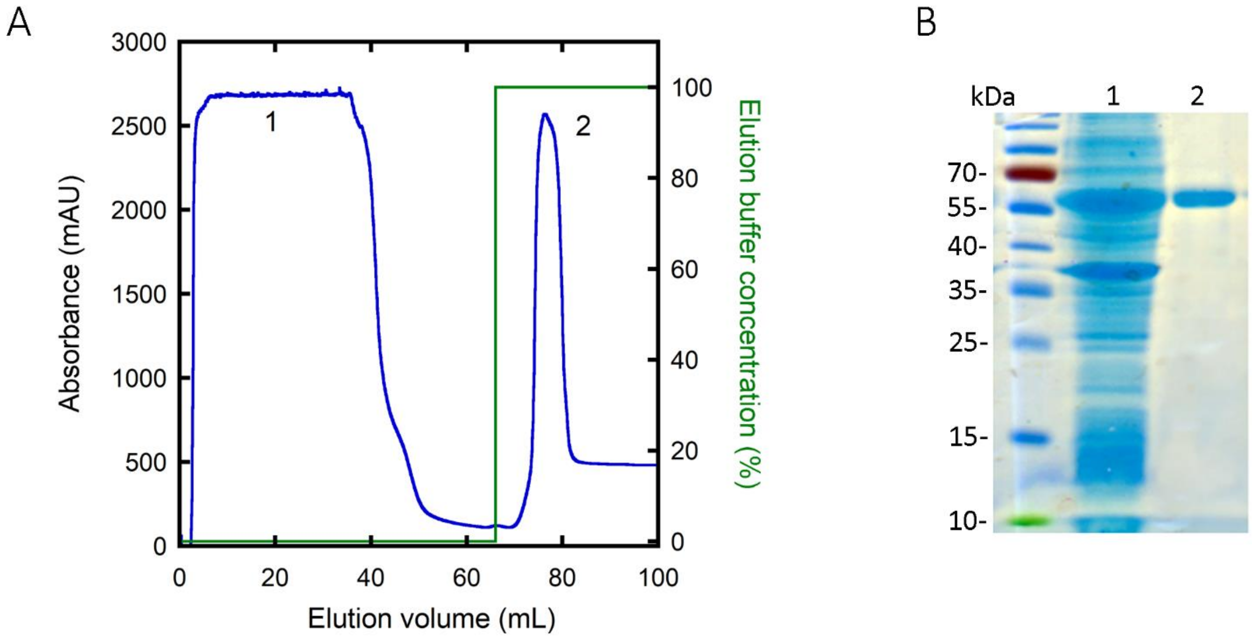

Figure S1: Purification of hPHGDH from *E. coli* crude extract. A) Chromatogram of the purification of His-tagged hPHGDH on a HiTrap chelating column. B) SDS-PAGE analysis of crude extract (fraction 1) and of purified hPHGDH (fraction 2).

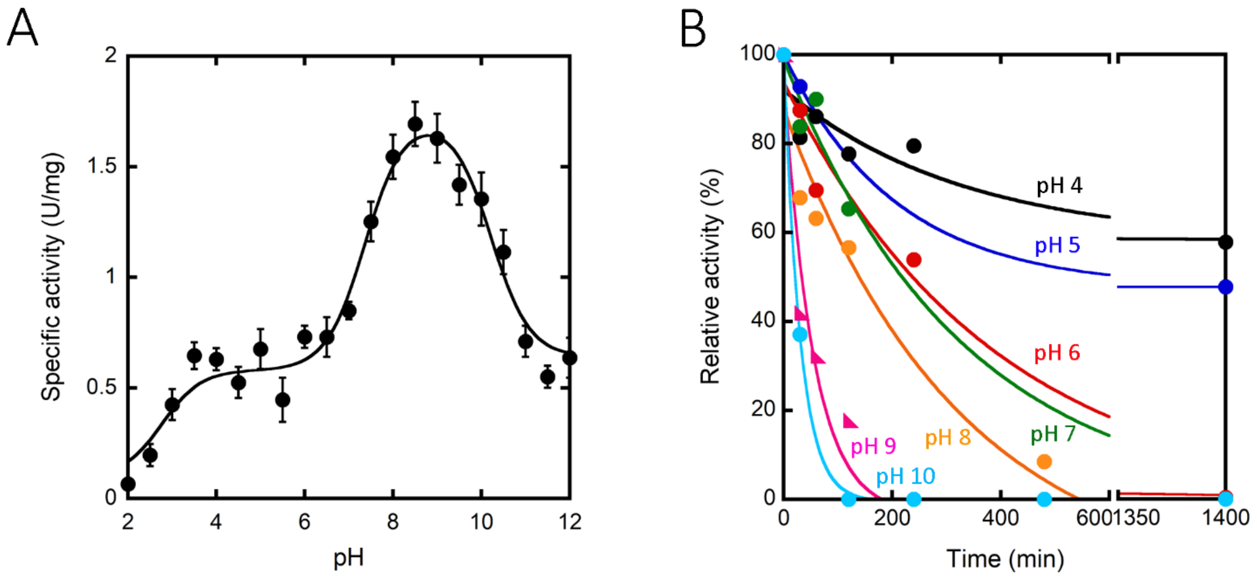

Figure S2: Effect of pH on activity (A) and stability (B) of hPHGDH. A) The activity was assayed at 2.4 mM 3PG and 0.12 mM NAD^+, following the incubation at 37 °C in a multi-component buffer at different pH values. Data points (mean ± SD, n = 3) were fitted according to the following equation [44]: $y = \frac{[a_1 + b_1 * 10^{(c1-x)}]}{1+10^{(c1-x)}} * \frac{[a_2 + b_2 * 10^{(c2-x)}]}{1+10^{(c2-x)}} * \frac{[a_3 - b_3 * 10^{(c3-x)}]}{1+10^{(c3-x)}}$ yielding apparent pK_{a1} = 2.8; pK_{a2} = 7.4; pK_{a3} = 10.2. B) Time course of hPHGDH residual activity following the incubation at different pH values (from 4 to 10, from top to bottom lines) and 37 °C. The residual activity of hPHGDH was assessed after 30, 60, 120, 240, 480 or 1400 min of incubation, and is related to the value determined at time = 0 min for each condition.

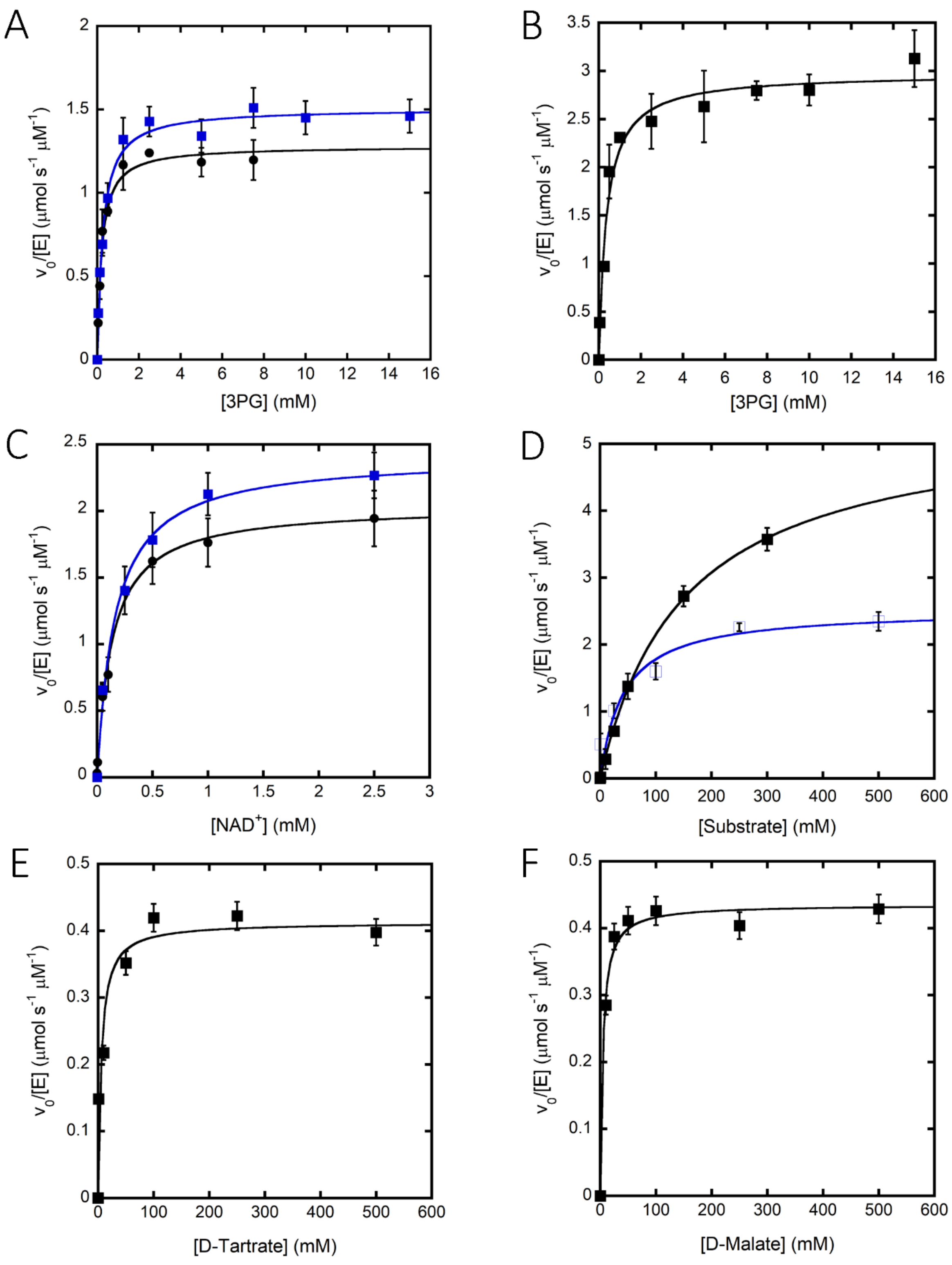

Figure S3: Kinetics of hPHGDH in the forward direction on different substrates. A) 3PG at 120 µM NAD^+ using the PSAT-coupled assay (circles) and the hydrazine (blue squares) assays, y = 1.282 x / (0.2518 + x), y = 1.5348 x / (0.3174 + x), respectively; B) 3PG at 1.5 mM NAD^+ using the hydrazine assay, y = 2.972 x / (0.3595 + x); C) NAD^+ at 2.5 mM 3PG using the PSAT-coupled assay (circles) and the hydrazine (blue squares) assays, y = 2.039 x / (0.13324 + x) and y = 2.4118 x / (0.16168 +

x), respectively; D) D-3-hydroxybutyrate (open blue symbols) and D-lactate (closed symbols) at 1.5 mM NAD^+, y = 2.527 x / (41.812 + x) and y = 5.407 x / (151.35 + x), respectively; E) D-tartrate at 1.5 mM NAD^+, y = 0.4125 x / (5.551 + x); F) D-malate at 1.5 mM NAD^+, y = 0.4351 x / (4.4767 + x). Data are shown as mean ± SD for three measurements; where not shown, bars are smaller than symbols used.

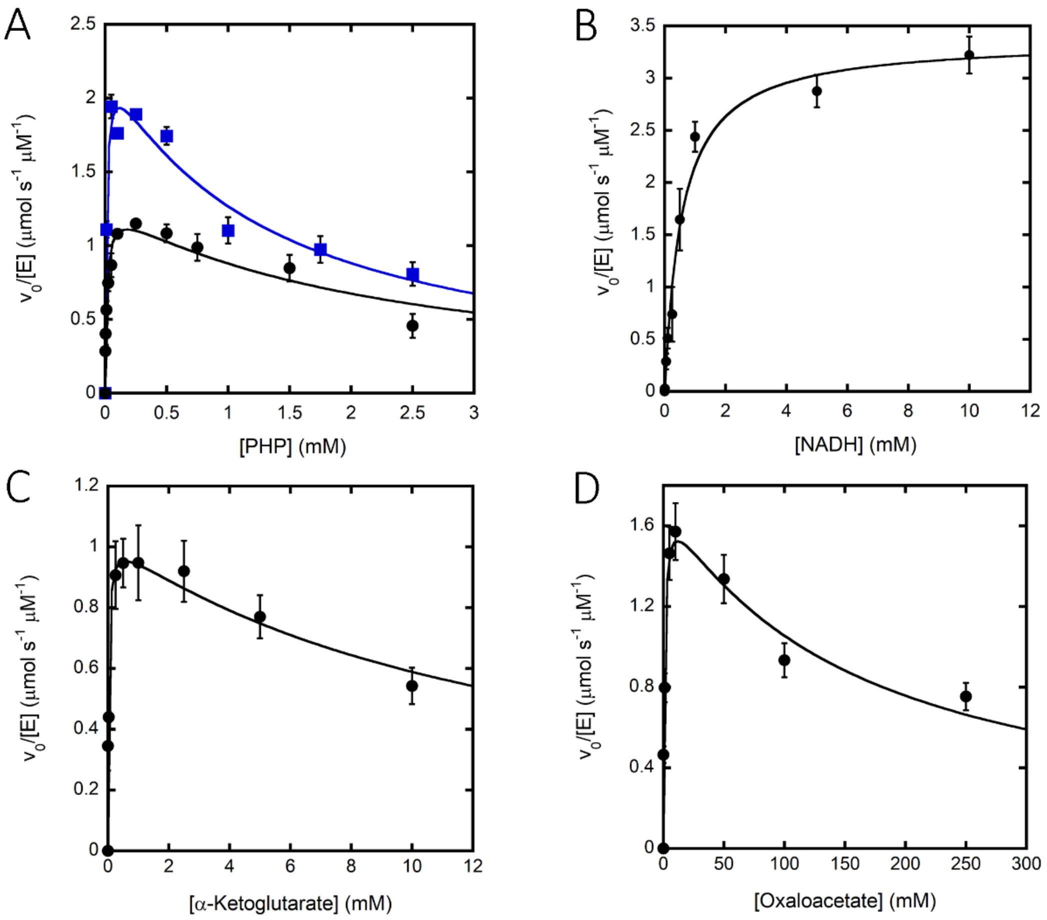

Figure S4. Kinetics of hPHGDH in the reverse direction on different substrates. A) PHP at 150 µM (circles) and 5 mM (blue squares) NADH, $y = 1.2871\ x / [(0.0141 + x + (x^2/2.2257)]$ and $y = 2.282\ x / [(0.0102 + x + (x^2/1.2646)]$, respectively; B) NADH at 0.5 mM PHP, $y = 3.3684\ x / (0.5587 + x)$; C) α-ketoglutarate at 5 mM NADH, $y = 1.0342\ x / [(0.02372 + x + (x^2/13.285)$; D) oxaloacetate at 5 mM NADH, $y = 1.7591\ x / [(0.9001 + x + (x^2/152.1)$. Data are shown as mean ± SD for three measurements. For PHP, α-ketoglutarate and oxaloacetate the experimental data were fit considering a substrate inhibition effect.

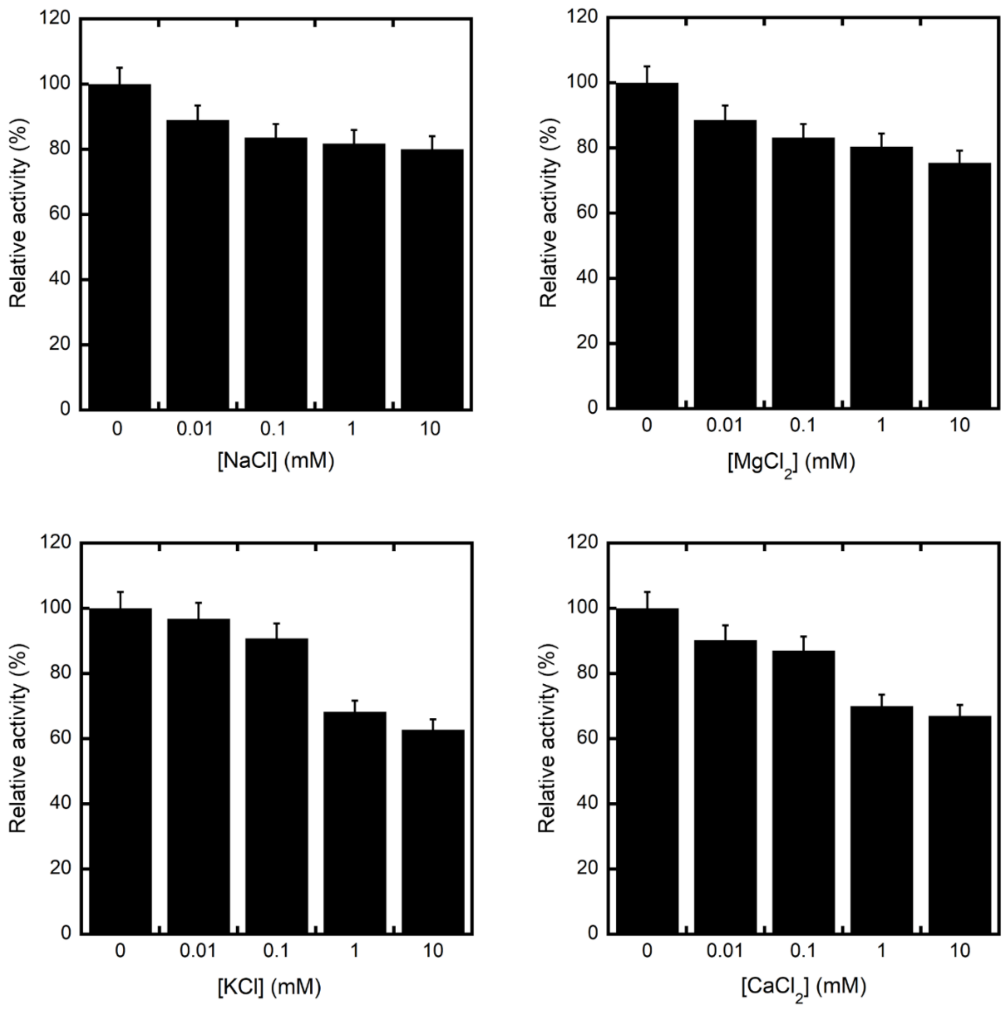

Figure S5: Effect of monovalent (left) and divalent (right) ions on hPHGDH activity at 2.4 mM 3PG and 0.12 mM NAD^+. Data are shown as mean ± SD for three measurements.

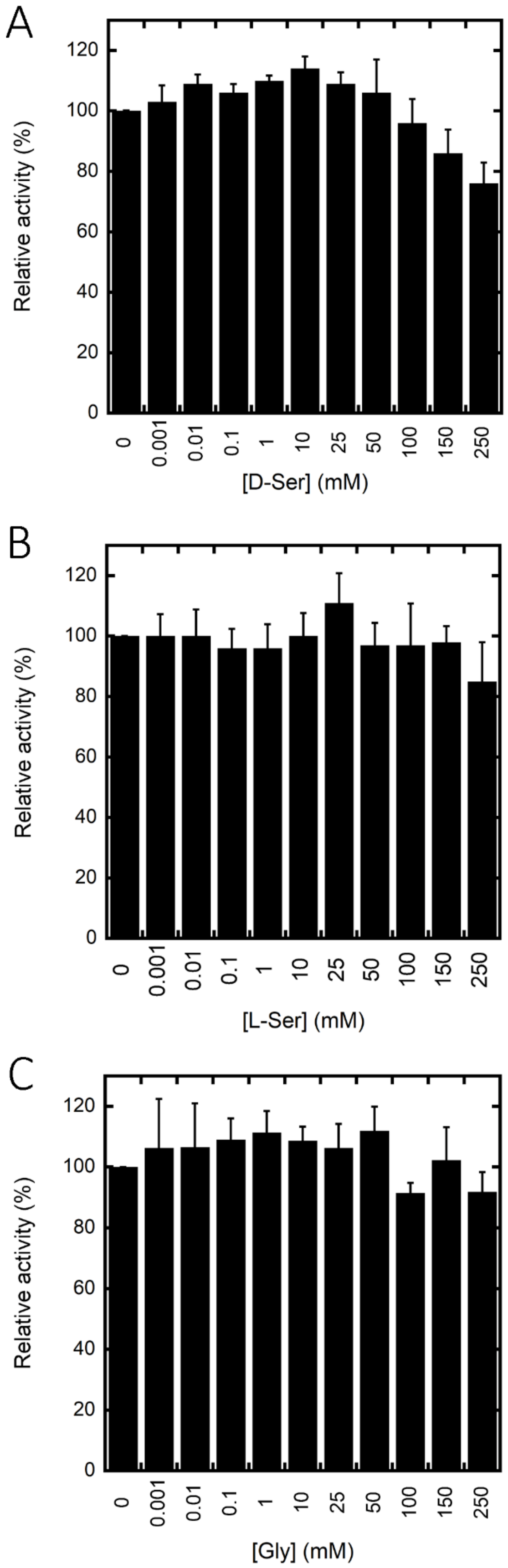

Figure S6: D-serine (A), L-serine (B) and glycine (C) marginally affect the activity of hPHGDH on 2.4 mM 3PG and 0.12 mM NAD^+. Data are shown as mean ± SD for three measurements.

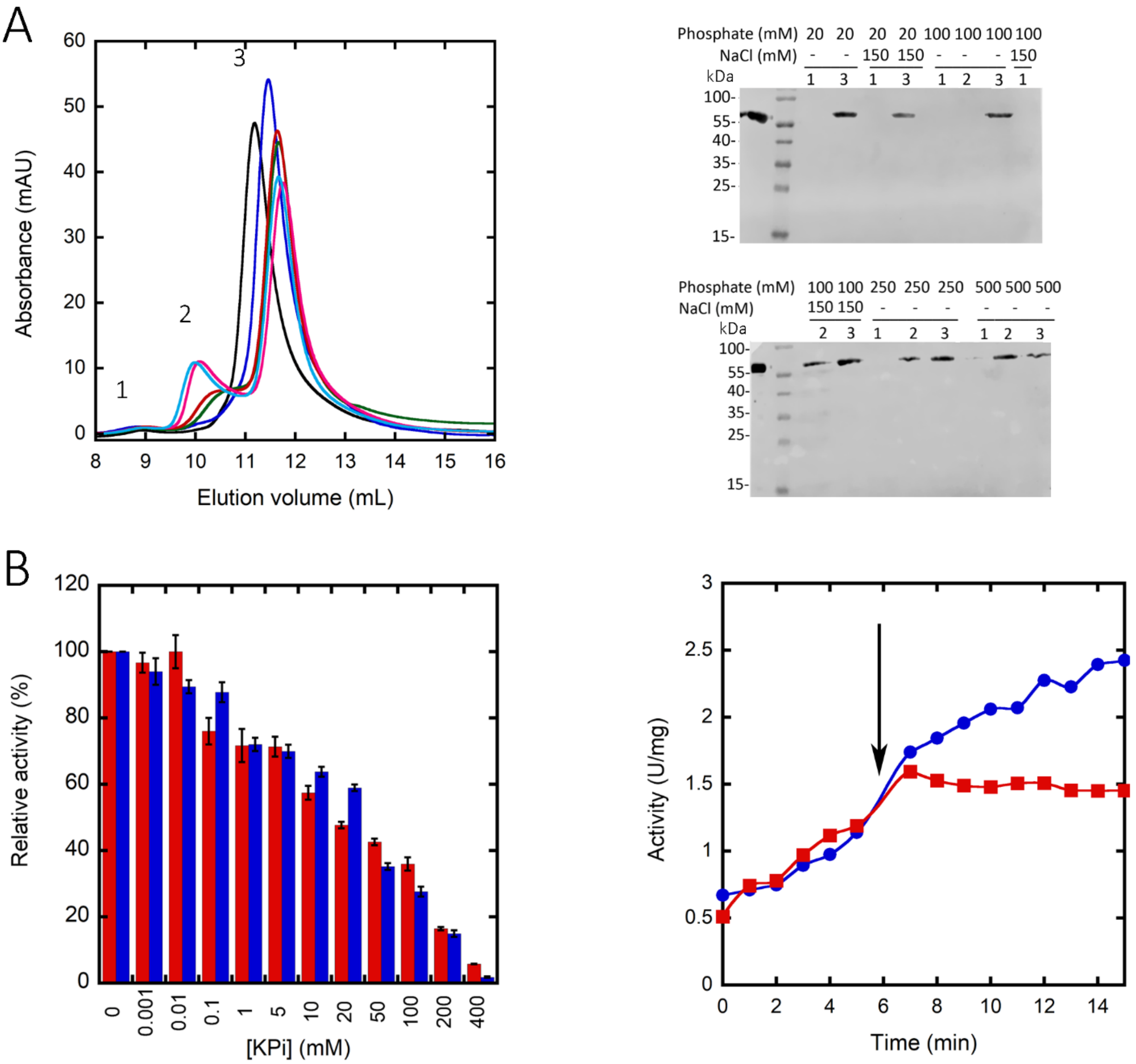

Figure S7: Effect of phosphate ions on hPHGDH properties. A) Oligomeric state. (Left) Size-exclusion chromatography elution profiles of hPHGDH at different concentrations of phosphate buffer: 20 mM (black), 20 mM + 150 mM NaCl (blue), 100 mM (green), 100 mM + 150 mM NaCl (red), 250 mM (magenta), and 500 mM (light blue). (Right) Western blot analysis of fractions eluted from size-exclusion chromatography and corresponding to different oligomerization states of the protein. Detection was performed using an anti-Histag antibody. B) Enzymatic activity. (Left) hPHGDH activity decreases at increasing potassium phosphate concentrations: forward reaction (blue bars), reverse reaction (red bars). Data are shown as mean ± SD for three measurements. (Right) Time course of hPHGDH activity: the standard enzymatic activity (blue) is quickly inhibited by adding 250 mM potassium phosphate (red, see black arrow).

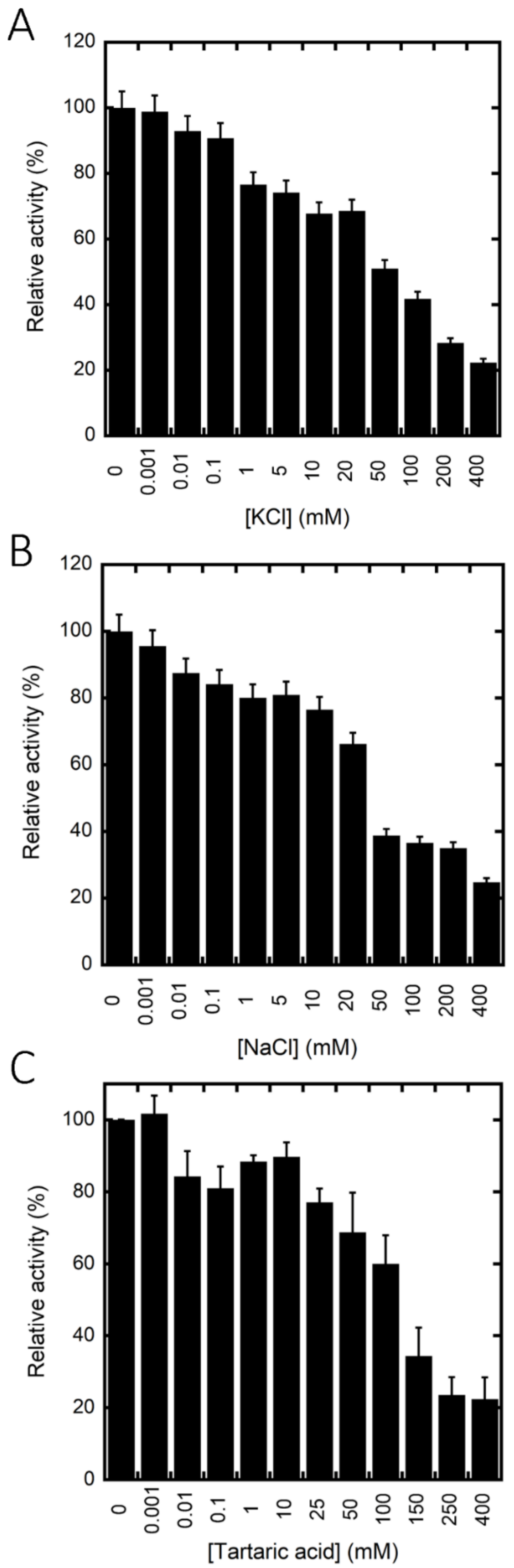

Figure S8: Effect of concentration of different salts on hPHGDH activity. The activity was assayed at 2.4 mM 3PG and 0.12 mM NAD^+ in presence of different amount of KCl (A), NaCl (B) and sodium tartrate (C). Data are shown as mean ± SD for three measurements.

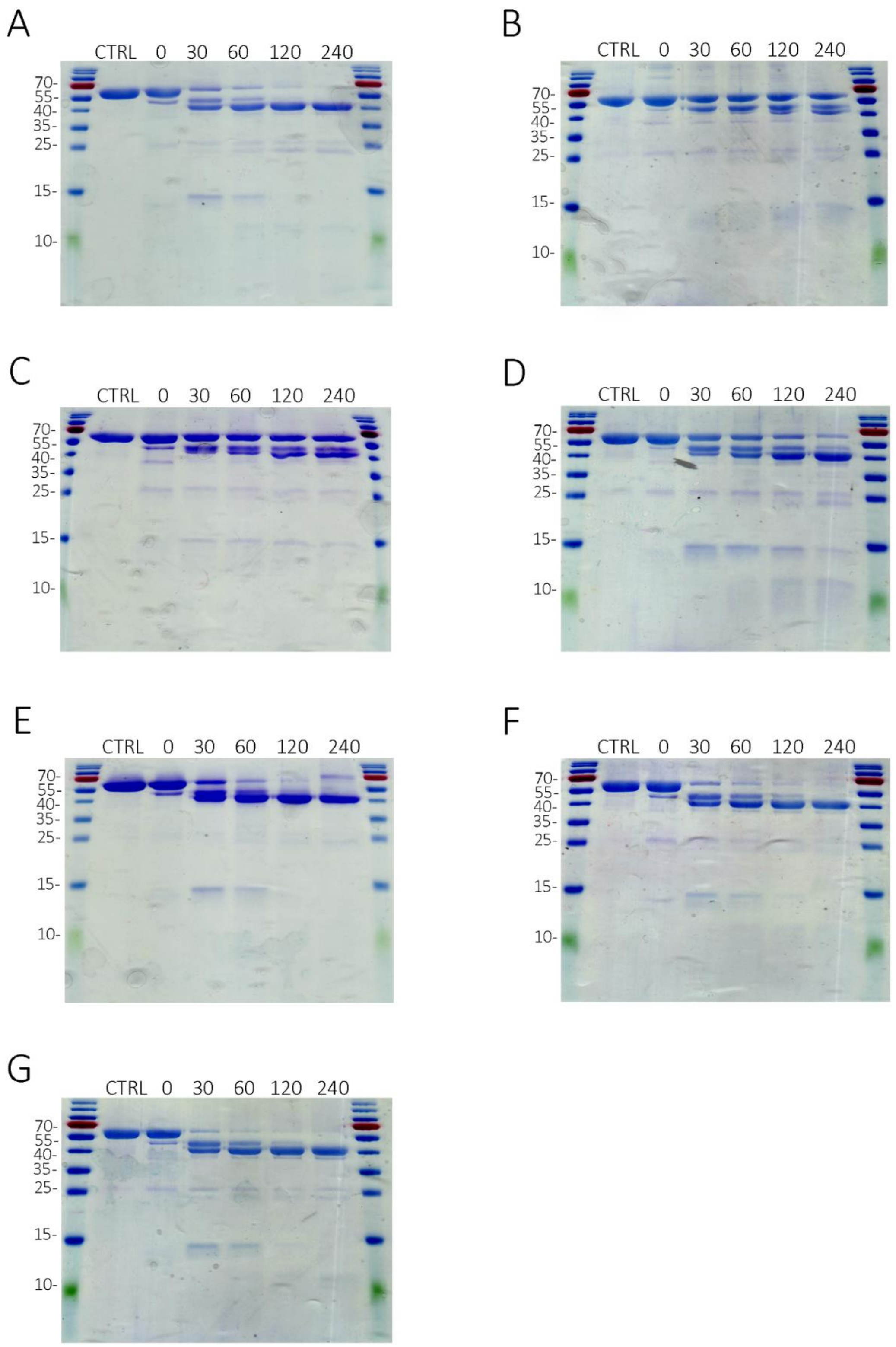

Figure S9: Limited proteolysis of hPHGDH evaluated by SDS-PAGE. 60 µg of hPHGDH (in 50 mM Hepes, pH 7, 100 mM NaCl, 10% glycerol, 1 mM EDTA and 5 mM 2-mercaptoethanol) was incubated at 37 °C with 5% (w/w) trypsin (panel A) and with: B) 250 mM potassium phosphate, C) 500 mM sodium tartrate, D) 0.1 mM NCT-503, E) 0.12 mM NAD^+, F) 2.4 mM 3PG, G) 0.12 mM NAD^+ and 2.4 mM 3PG.
